# Infantile epileptic spasms syndrome as a new phenotype in TOP2B deficiency caused by a *de novo* variant: a case report and literature review

**DOI:** 10.3389/fped.2025.1542268

**Published:** 2025-06-27

**Authors:** Guo-qin Zhu, Yao Yao, Ling-yun Yang, Ying Hua, Guo-min Li

**Affiliations:** ^1^ Department of Nephrology, Rheumatology and Immunology, Children’s Hospital of Jiangnan University, Wuxi, Jiangsu, China; ^2^ Department of Nephrology, Rheumatology and Immunology, Wuxi Children's Hospital, Wuxi, Jiangsu, China; ^3^ Department of Neurology, Children’s Hospital of Jiangnan University, Wuxi, Jiangsu, China

**Keywords:** case report, type II topoisomerase β, West syndrome, B-cell immunodeficiency, neurodevelopmental disorders

## Abstract

**Background:** Type II DNA topoisomerases (EC5.99.1.3) are enzymes that catalyze topological changes during DNA replication and gene transcription in an ATP-dependent manner. Vertebrates have two isoforms: topoisomerase II*α* and *β*. Type II topoisomerase *β* is encoded by *TOP2B*. For *TOP2B*, a number of germline pathogenic variants have been identified as causative for human diseases, including Hoffman syndrome, ablepharon-macrostomia syndrome with immunodeficiency, B-cell immunodeficiency, distal limb anomalies, and urogenital malformations syndrome. To date, only 14 patients with the above diseases from seven families have been reported in PubMed. Herein, we describe an additional case of a child who presented with “infantile epileptic spasms syndrome” (IESS) as the first symptom, B-cell immunodeficiency, dysmorphic facial features, and a pathogenic variant in *TOP2B*. The c.1901A > G variant in *TOP2B* is new to our study, which further enriches the genotype of TOP2B deficiency. Our patient manifested as a typical triad: infantile spasms, hypsarrhythmia on electroencephalogram, and developmental arrest at the age of 7 months. Although epilepsy and neurodevelopmental disorders have been reported in patients with TOP2B deficiency, typical IESS has not been described previously. IESS in our patient further expands the phenotype of *TOP2B*. The patient was started on monthly intravenous immunoglobulin replacement therapy after being diagnosed with TOP2B deficiency and since then has not suffered from severe infections. TOP2B deficiency is a group of heterogeneous diseases, which is ultrarare. The results from our study extend the phenotype and genotype spectrum of TOP2B deficiency. *TOP2B* may be a causative gene for IESS.

## Introduction

DNA topoisomerases are essential enzymes required for the relaxation of topological stress during DNA replication and gene transcription (1, 2). Mammalian proteins have two isoforms: type II topoisomerase alpha and type II topoisomerase beta. Type II topoisomerase β, encoded by *TOP2B*, was first reported in 1987 by Fred Drake's group at Smith Kline French (SKF) (3). In mammalian cells, *TOP2B* is expressed in proliferative and terminally differentiated postmitotic cells (4, 5). A microarray analysis of *TOP2B* expression in primary human cells (https://BioGPS.org) revealed broad expression and high expression in CD34^+^ bone marrow cells (6). *TOP2B* plays a critical role in B-cell development and dominant pathogenic variants in *TOP2B* have been shown to lead to B-cell deficiency (7, 8). However, a transcriptome analysis of human tissues via the GTEx portal (https://www.gtexportal.org) has also revealed a high expression of *TOP2B*, with the highest expression in the cerebellum (9). *TOP2B* essentially affects neuronal differentiation, survival, DNA repair, and neurite outgrowth and is involved in brain development and neural differentiation. Recently, several *de novo* missense *TOP2B* variants have been identified in patients with neurodevelopmental disorders (NDDs) (10, 11).

West syndrome (WS) is characterized by infantile spasms, hypsarrhythmia on electroencephalogram (EEG), and developmental arrest or regression (12, 13). WS has recently been reclassified into infantile epileptic spasms syndrome (IESS), a devastating developmental epileptic encephalopathy (DEE) consisting of epileptic spasms, as well as one or both of developmental regression or stagnation and hypsarrhythmia on EEG (14–16). To date, over 28 copy number variants (CNVs) and 70 single-gene pathogenic variants related to IECSS have been discovered (17). However, *TOP2B* was not included in the list of genes associated with IESS (12, 17). Here, we report a Chinese girl who presented with IESS and B-cell deficiency caused by a *de novo TOP2B* variant.

## Case presentation

**Figure 1:**
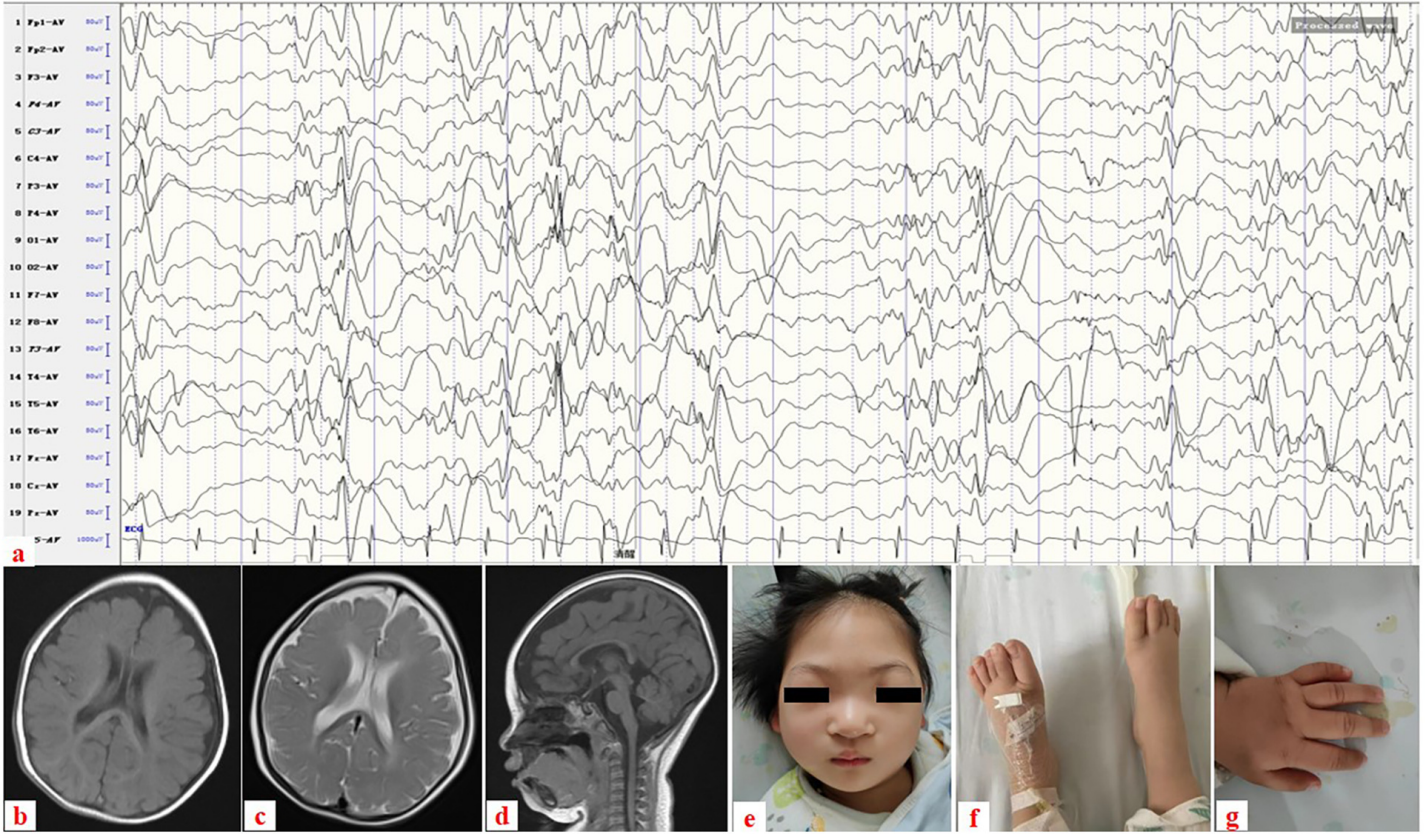
**(a)** Electroencephalogram showing hypsarrhythmia. **(b–d)** Brain MRI taken at 7 months’ old: **(b)** axial image in T1-weighted image, **(c)** axial image in T2-weighted image, and **(d)** sagittal image in T1-weighted image reveal cerebral atrophy with subsequent enlargement of the subarachnoid spaces. **(e–g)** Clinical photograph taken at 1 year and 6 months’ old: **(e)** Dysmorphic facial features with microcephaly and ocular hypertelorism, **(f)** normal feet, and **(g)** normal left hand.

The patient was a 2-year-old Chinese girl who was the first child of a non-consanguineous couple. She was born by cesarean section due to prolonged labor and had no asphyxia, with a birth weight of 3.25 kg. At 7 months of age, she could not roll over or reach out to objects. She was admitted to the Department of Neurology at our hospital because of axial spasms in clusters at the age of 9 months. A physical examination revealed that she was hypotonic but had normal muscle power and deep tendon reflexes. Her head circumference, length, and weight were 39 cm (less than the 3rd percentile, 41.7 cm), 68 cm (3rd–10th percentile), and 7.3 kg (75th–90th percentile), respectively. An EEG was performed and it revealed hypsarrhythmia (Figure 1a). MRI scans of the brain revealed marked cerebral atrophy with subsequent enlargement of the subarachnoid spaces (Figures 1b–d). She was diagnosed with IESS based on infantile spasms, hypsarrhythmia, and developmental arrest. Despite the use of different combinations of anticonvulsants (vigabatrin, lamotrigine, and vitamin B6), her epilepsy could not be completely controlled.

**Figure 2:**
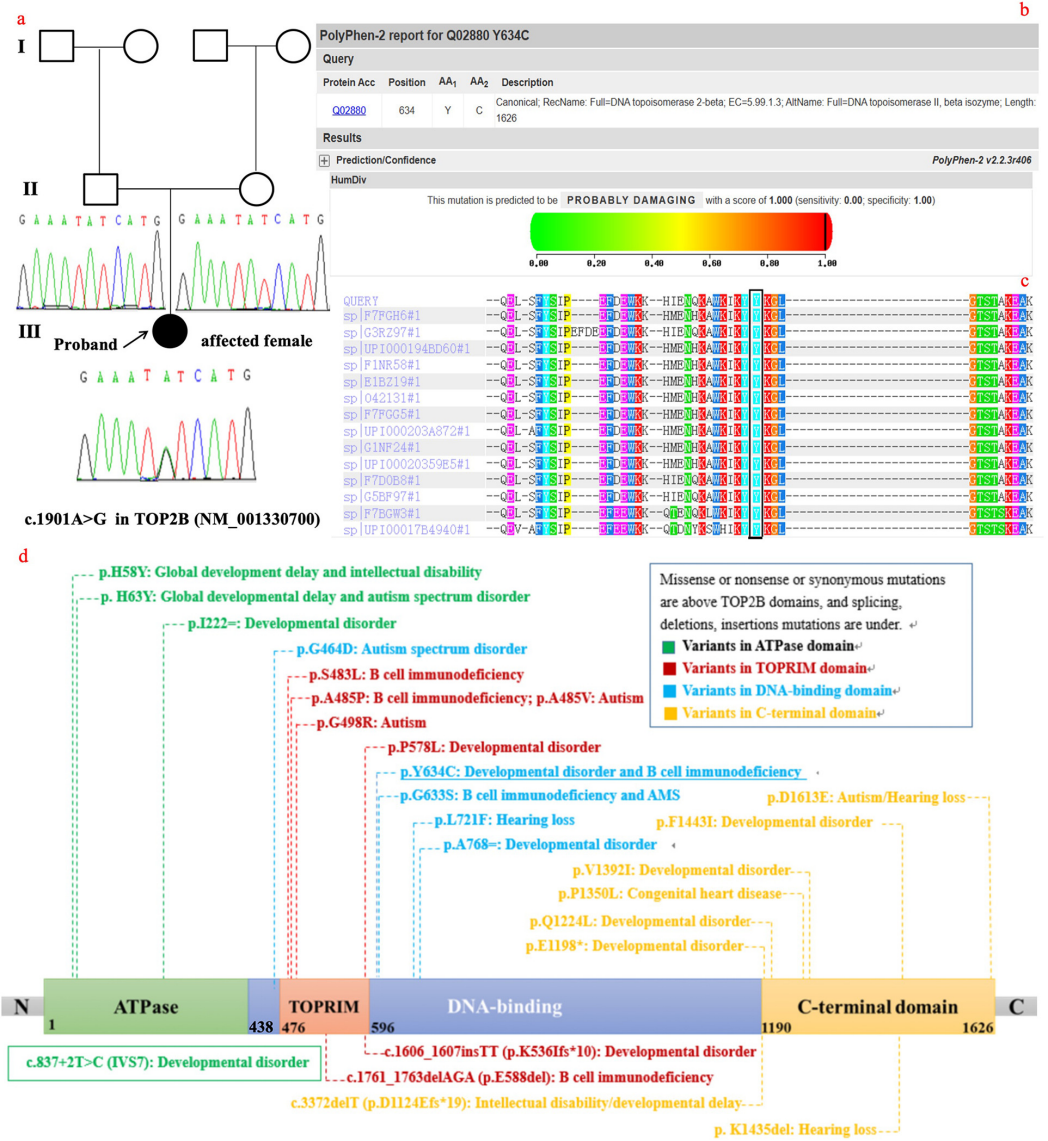
**(a)** familial pedigrees and sanger sequencing of *TOP2B*. **(b)** The p.Y634C variant is predicted to be “probably damaging” by Polyphen2. **(c)** Alignment of the mutated p.Y634C TOP2B protein with different species shows the complete conservation of the amino acid. **(d)** The TOP2B protein consists of 1,621 amino acids that form an ATPase domain, TOPRIM domain and DNA-binding region, and a C-terminal region, and all variants reported from other studies are located in different structural domains.

She was referred to our department for evaluation because of recurrent infections and hypogammaglobulinemia at the age of 1 year. A clinical examination revealed dysmorphic facial features with microcephaly and ocular hypertelorism (Figure 1e), global developmental delay with severe muscular hypotonia, significantly decreased head control, and only sparse spontaneous movements. The distal limb was normal (Figures 1f,g). Ocular fixation was not evident. She had poor eye contact and no eye fixation to moving objects. Laboratory findings revealed severe hypogammaglobulinemia: IgG, 1.8 g/L (normal: 5.4–15.1 g/L); IgM, 0.12 g/L (normal: 0.48–2.31 g/L); and IgA, 0.0 g/L (normal: 0.52–2.74 g/L). Total white blood cell count was normal, with normal absolute neutrophil and lymphocyte counts. However, a lymphocyte subset analysis using flow cytometry revealed undetectable CD19^+^ B lymphocytes (Table 1). Two variants (Table 2) were detected using tri-based whole-exome sequencing (WES): a heterozygous *de novo* c.1901A > G (p.Y634C) variant in *TOP2B* (NM_001330700) and a homozygous c.3163G > A (p.V1055I) variant in *VARS1* (NM_006295). The c.1901A > G variant in *TOP2B* in the patient was confirmed using Sanger sequencing and was not detected in her parents (Figure 2a). The c.1901A > G (p.Y634C) variant in *TOP2B* was evaluated as a pathogenic variant through the American College of Medical Genetics and Genomics/Association for Medical Pathology (ACMG/AMP) variant classification guidelines, whereas the c.3163G > A (p.V1055I) variant is of uncertain significance (VUS). The c.1901A > G variant was not found in ExAC (https://gnomad.broadinstitute.org/) or 1000G (http://browser.1000genomes.org). The c.1901A > G variant is predicted to be “probably damaging” by Polyphen2 with a score of 1.0 (sensitivity: 0.0; specificity: 1.0; Figure 2b), and MutationTaster2021 software predicted this variant to be disease causing. In addition, alignment of the mutated p.Y634C TOP2B protein with different species revealed the complete conservation of the amino acid (Figure 2c). However, both the heterozygous and the homozygous c.3163G > A variants have been found in both ExAC (https://gnomad.broadinstitute.org/) and 1000G (http://browser.1000genomes.org). The c.3163G > A variant was predicted to be “possibly damaging” by Polyphen2 with a score of 0.69 (sensitivity: 0.86; specificity: 0.92), and MutationTaster2021 software predicted this variant to be a polymorphism. The patient was started on monthly intravenous immunoglobulin (IVIG) replacement therapy after being diagnosed with TOP2B deficiency and since then has not suffered from severe infections.

**Table 1:** Lymphocyte subsets in the patient.

Classification of lymphocyte	Lymphocyte subsets	Surface molecules	Denominator	Test value (%)	Reference range (%)
T cells	Total of T cells	CD3^+^	Lymphocyte	94.47	60.15–72.29
	CD4^+^ T cells	CD3^+^CD4^+^	Lymphocyte	53.17	35.23–51.41
	CD8^+^ T cells	CD3^+^CD8+	Lymphocyte	39.21	14.11–27.77
	Double negative T cells	CD3^+^CD4^−^CD8^−^	CD3^+^T cell	2.31	—
	TCRαβ^+^ double positive T cells	CD3^+^CD4^−^CD8^−^ TCRαβ^+^	CD3^+^T cell	0.36	0.57–1.53
	TCR*γδ*^+^ T cells	CD3^+^TCRγδ+	Lymphocyte	2.26	3.80–9.20
T lymphocyte subsets	CD4^+^ initial T cells	CD3^+^CD4^+^CD27^+^CD45RA^+^	CD4^+^T cells	81.73	65.58–87.86
	CD4^+^ central memory T cells	CD3^+^CD4^+^CD27^+^CD45RA^−^	CD4^+^T cells	17.35	11.74–32.72
	CD4^+^ effector memory T cells	CD3^+^CD4^+^CD27^−^CD45RA^−^	CD4^+^T cells	0.87	0.42–2.26
	CD4^+^ end-stage T cells	CD3^+^CD4^+^CD27^−^CD45RA^+^	CD4^+^T cells	0.05	0.00–1.07
	CD8^+^ initial T cells	CD3^+^CD8^+^CD27^+^CD45RA^+^	CD8^+^T cells	91.83	53.16–90.14
	CD8^+^ central memory T cells	CD3^+^CD8^+^CD27^+^CD45RA^−^	CD8^+^T cells	8.06	7.09–31.70
	CD8^+^ effector memory T cells	CD3^+^CD8^+^CD27^−^CD45RA^−^	CD8^+^T cells	0.07	0.18–7.06
	CD8^+^ end-stage T cells	CD3^+^CD8^+^CD27^−^CD45RA^+^	CD8^+^T cells	0.03	0.12–20.70
B lymphocyte subsets	CD19^+^ B cell	CD19^+^	Lymphocyte	0.15	16.57–27.65
	Initial B cell	CD19^+^CD27^−^IgD^+^	CD19^+^B cells	69.33	79.08–93.04
	Marginal zone B cell	CD19^+^CD27^+^IgD^+^	CD19^+^B cells	18.67	3.00–10.70
	Memory B cell	CD19^+^CD27^+^	CD19^+^B cells	2.67	1.24–7.81
	Transitional B cell	CD19^+^CD38^high+^CD24^high+^	CD19^+^B cells	5.33	5.84–18.76
	Plasmablasts	CD19^+^CD38^high+^	CD19^+^B cells	2.67	0.44–7.40

**Table 2:** Variants in the patients.

Gene	Location in Chr	Reference sequence	Exon	cDNA	Protein	Variant status	ACMG grade	MAF	Inheritance patterns	Diseases/phenotype	Variant origin
*TOP2B*	Chr3-25670343-25670343	NM_01330700	15	c.1901A > G	p.Y634C	Het	Pathogenic: PS2 + PM1 + PM2 PP2 + PP3	0	AD	BILU syndrome	*De novo*
										Hoffman syndrome	
										AMS with immunodeficiency	
*VARS1*	Chr6-31747510-31747510	NM_006295	27	c.3163G > A	p.V1055I	Hom	Uncertain significance: PM1 + PP2 + BS1	0.0136	AR	Neurodevelopmental disorder	Parents
										Microcephaly	
										Seizures cortical atrophy	

PS2, *de novo* (both maternity and paternity confirmed) in a patient with the disease and no family history; PM1, located in a mutational hot spot and/or critical and well-established functional domain (e.g., active site of an enzyme) without benign variation; PM2, absent from controls (or at extremely low frequency if recessive) (table 6) in Exome Sequencing Project, 1,000 Genomes Project, or Exome Aggregation Consortium; PP2, missense variant in a gene that has a low rate of benign missense variation and in which missense variants are a common mechanism of disease; PP3, multiple lines of computational evidence support a deleterious effect on the gene or gene product (conservation, evolutionary, splicing impact, etc.); BS1, allele frequency is greater than expected for disorder; AD, autosomal-dominant inheritance; AR, autosomal recessive inheritance; AMS, ablepharon-macrostomia syndrome; BILU, B-cell immunodeficiency, distal limb anomalies and urogenital malformations; MAF, majority allele frequency.

This study was approved by the Ethics Committee at the Children's Hospital of Jiangnan University, and written informed consent was obtained from the parents of the patient.

## Discussion and conclusions

*TOP2B* encodes a DNA topoisomerase, which is involved in processes such as chromosome condensation, chromatid separation, and the relief of torsional stress that occurs during DNA transcription and replication (5). For *TOP2B*, a number of germline pathogenic variants have been identified as causative factors for human diseases. The diseases associated with ***T****OP2B* (**DAT**) include four clinical entities: (1) Hoffman syndrome; (2) B-cell immunodeficiency, distal limb anomalies, and urogenital malformations (BILU) syndrome (MIM #609296); (3) ablepharon-macrostomia syndrome (AMS; MIM #200110) with immunodeficiency; and (4) autosomal-dominant hereditary hearing loss (18–25). To date, only 14 patients with Hoffman syndrome, BILU, and AMS from seven families have been reported in PubMed (10, 11, 18–24). Therefore, this condition is very rare, with a prevalence of <1/1,000,000 (26). Herein, we describe an additional case of a child who presented with WS as the first symptom, B-cell immunodeficiency, and dysmorphic facial features. A c.1901A > G variant in *TOP2B* was detected by trio-WES in the patient. The c.1901A > G variant was evaluated to be pathogenic through ACMG/AMP variant classification guidelines. The c.1901A > G variant is predicted to be “probably damaging” by Polyphen2, and MutationTaster2021 software was used to predict this variant to be disease causing. In addition, alignment of the mutated p.Y634C *TOP2B* protein with different species revealed the complete conservation of the amino acid. Although functional studies of the variant were not performed, the above bioinformatics analysis suggested that the variant is pathogenic. A total of 23 variants were reported in TOP2B including 15 missense, 2 synonymous, 1 non-sense, 1 splicing, 3 small deletions, and 1 small insertion mutation in the human gene mutation database (HGMD) professional 2023.4 (Figure 2d) (27). The c.1901A > G variant is new in our study, further enriching the genotype of TOP2B.

A homozygous c.3163G > A variant in *VARS1* was found by trio-WES in the patient and evaluated to be VUS through ACMG/AMP variant classification guidelines. The c.3163G > A variant is predicted to be “possibly damaging” by Polyphen2 and MutationTaster2021 software was used to predict this variant to be polymorphic. Thus, the homozygous c.3163G > A variant in *VARS1* may not lead to neurological symptoms or signs in the patient.

Eukaryotic TOP2B proteins have evolutionarily conserved domain structures: they consist of 1621 amino acids that form an ATPase domain, a central catalytic core domain, and a C-terminal region (28, 29). The central catalytic core domain of eukaryotic TOP2B proteins, also known as the breakage-reunion domain, has a TOPRIM domain and a DNA-binding region and has catalytic properties (30). A total of 24 variants (including those in our study) are located in the N-terminal ATPase domain, the central catalytic core domain, and the C-terminal region (Figure 2d). Variants in the N-terminal ATPase domain and the C-terminal region are associated with developmental disorders, whereas variants in the central catalytic core domain are related to B-cell deficiency. Variants reported in patients with hearing loss are in both the central catalytic core domain and the C-terminal region (25). The variant in this study was in the central catalytic core domain, and our patient had both severe developmental disorders and B-cell deficiency. Our patient manifested as a typical triad: infantile spasms, hypsarrhythmia on EEG, and developmental arrest at the age of 7 months, which meets the diagnostic criteria for IESS. Developmental arrest included motor and speech developmental disorders in the patient. In addition to infantile spasms and developmental arrest, neurological involvement includes growth delay (short stature), intellectual disability, hypotonia, and autistic features. Among patients in reported studies, those with DAT have the youngest onset age. While neurodevelopmental disorders are frequently reported in patients with DAT, epilepsy is less commonly observed in this population (10, 11, 24). Tonic-clonic seizures were reported in a 6-year-3-month-old child with DAT (11); the clinical presentation did not fulfill the diagnostic criteria for IESS. IESS in this patient further expands the phenotype of TOP2B deficiency. Various combinations of anticonvulsants, including vigabatrin, lamotrigine, and vitamin B6, were administered to the patient, but her seizures remained refractory. Adrenocorticotropic hormone (ACTH) and oral corticosteroids were not used as first-line treatments in this case for two reasons: first, her comorbid immunodeficiency increased the risk of severe infections from immunosuppressive therapy; second, her epilepsy was genetically determined and often showed poor response to anticonvulsant medications.

DAT is a group of heterogeneous diseases, including Hoffman syndrome, BILU, AMS with immunodeficiency, and autosomal-dominant hereditary hearing loss. This heterogeneity may be due to variants located in different structural domains. An increasing number of studies indicate that TOP2B deficiency is associated with NDD.

*TOP2B* may be a causative gene for IESS.

## Limitations

Although the TOP2B variant was predicted to be pathogenic through bioinformatics analysis, functional studies were not performed to validate this finding; due to the presence of central nervous system phenotypes (e.g., epilepsy and developmental delay), mitochondrial DNA testing was not conducted, leaving the potential contribution of mitochondrial gene variants unexamined. Further investigations are planned to address these limitations, including functional validation of the TOP2B variant and mitochondrial genome sequencing, to enhance the reliability of the current findings.

## Data Availability

The original contributions presented in the study are included in the article/Supplementary Material, further inquiries can be directed to the corresponding authors.
